# Amino Acids and IGF1 Regulation of Fish Muscle Growth Revealed by Transcriptome and microRNAome Integrative Analyses of Pacu (*Piaractus mesopotamicus*) Myotubes

**DOI:** 10.3390/ijms23031180

**Published:** 2022-01-21

**Authors:** Bruno Oliveira Silva Duran, Bruna Tereza Thomazini Zanella, Erika Stefani Perez, Edson Assunção Mareco, Josefina Blasco, Maeli Dal-Pai-Silva, Daniel Garcia de la serrana

**Affiliations:** 1 Department of Histology, Embryology and Cell Biology, Institute of Biological Sciences, Federal University of Goiás (UFG), Goiânia 74690-900, GO, Brazil; brunoduran@ufg.br; 2 Department of Structural and Functional Biology, Institute of Biosciences, São Paulo State University (UNESP), Botucatu 18618-689, SP, Brazil; bruna.zanella@unesp.br (B.T.T.Z.); erika.perez@unesp.br (E.S.P.); maeli.dal-pai@unesp.br (M.D.-P.-S.); 3 Environment and Regional Development Graduate Program, University of Western São Paulo (UNOESTE), Presidente Prudente 19050-680, SP, Brazil; edsonmareco@gmail.com; 4 Department of Cell Biology, Physiology and Immunology, Faculty of Biology, University of Barcelona, 08028 Barcelona, Spain; jblasco@ub.edu

**Keywords:** muscle growth, cell culture, amino acids, IGF1, omics

## Abstract

Amino acids (AA) and IGF1 have been demonstrated to play essential roles in protein synthesis and fish muscle growth. The myoblast cell culture is useful for studying muscle regulation, and omics data have contributed enormously to understanding its molecular biology. However, to our knowledge, no study has performed the large-scale sequencing of fish-cultured muscle cells stimulated with pro-growth signals. In this work, we obtained the transcriptome and microRNAome of pacu (*Piaractus mesopotamicus*)-cultured myotubes treated with AA or IGF1. We identified 1228 and 534 genes differentially expressed by AA and IGF1. An enrichment analysis showed that AA treatment induced chromosomal changes, mitosis, and muscle differentiation, while IGF1 modulated IGF/PI3K signaling, metabolic alteration, and matrix structure. In addition, potential molecular markers were similarly modulated by both treatments. Muscle-miRNAs (*miR-1*, *-133*, *-206* and *-499*) were up-regulated, especially in AA samples, and we identified molecular networks with omics integration. Two pairs of genes and miRNAs demonstrated a high-level relationship, and involvement in myogenesis and muscle growth: *marcksb* and *miR-29b* in AA, and *mmp14b* and *miR-338-5p* in IGF1. Our work helps to elucidate fish muscle physiology and metabolism, highlights potential molecular markers, and creates a perspective for improvements in aquaculture and in in vitro meat production.

## 1. Introduction

The skeletal muscle in teleost fish represents up to 60% of its total body mass and is the most abundant tissue, with a set of characteristics necessary for fish physiology and metabolism, and with great importance for aquaculture industry [1,2]. Muscle growth is a multifactorial process regulated by extrinsic and intrinsic signals. Extrinsic factors include nutrient availability, temperature, salinity, oxygenation, photoperiod, pH, and water flow [3]. Intrinsic signals include transcription factors (such as myogenic regulatory factors, MRFs), hormones, cytokines, and growth factors. These inputs can shift the balance between protein synthesis and degradation pathways, promoting protein accretion by favoring protein synthesis, and therefore muscle growth [4]. Protein synthesis is strongly regulated by the IGF/PI3K/MTOR axis, while protein degradation is mediated by the ubiquitin-proteasome, calpain/calpastatin and autophagic-lysosomal systems [5,6].

Some of the main factors that regulate protein synthesis are IGFs (insulin-like growth factors), together with their own receptors (IGFR—insulin-like growth factor receptor) and binding proteins (IGFBP—insulin-like growth factor-binding protein) [7]. The IGFs are circulating peptides that comprise mostly two variants, IGF1 and IGF2, with roles in muscle cell viability, the proliferation and differentiation of myoblasts, and hypertrophy and repair after muscle injury and exercise [8,9,10]. The IGF1 is one of the most studied and characterized growth factors that promote muscle growth. When binding to its receptor (IGF1R), IGF1 triggers a phosphorylation cascade, promoting the activation of PI3K (phosphatidylinositol-3-kinase), which is necessary to produce phosphatidylinositol-3,4,5-triphosphate. This component recruits the AKT (protein kinase B), which subsequently activates MTOR (mechanistic target of rapamycin kinase) by phosphorylation [4]. The MTOR integrates endocrine signals, regulates cell cycle, gene transcription, cytoskeletal organization, and protein synthesis. Besides, the pathways involved in protein synthesis control can be activated by signals other than growth factors. Several studies have shown that amino acids promote the phosphorylation of MTOR [11,12] and regulate the transcription and activation of components of the IGF system [13,14] on their own. These studies support the existence of an independent route stimulated by amino acids to promote muscle growth in teleost fish. Given the roles of the IGF system and amino acids in promoting protein synthesis and muscle formation, the comprehension of their effects is beneficial for understanding muscle metabolism and for aquaculture.

The microRNAs (miRNAs) also play a fundamental role in controlling the progression of the myogenic program and the determination of the muscle fiber phenotypes [15,16]. The miRNAs correspond to a class of small non-coding RNAs of which the main function is the post-transcriptional regulation of genes, promoted by the translation inhibition or decay of messenger RNAs (mRNAs) [16,17,18]. miRNAs regulate their targets in a combinatorial pattern, increasing the complexity and regulatory potential of gene expression, with most of them able to finely regulate signaling pathways and common biological functions [19,20]. miRNAs in teleost fish are involved in embryogenesis and several developmental and physiological processes in different tissues [21,22,23,24,25,26,27,28]. Both muscle formation and growth are regulated by several miRNAs, with some of them considered as muscle-specific with unique or high expression in skeletal muscles, such as *miR-1*, *miR-133*, *miR-206* or *miR-499*. These miRNAs apply a high degree of control over the different phases of myogenesis, including myoblast proliferation, myotube formation, fiber type specification, and muscle regeneration [29,30,31,32], orchestrating the fate and phenotype of muscle cells.

Fish myoblast cell cultures are a very powerful tool to study all of these molecular networks and signals regulating myogenesis and muscle growth [33,34,35,36,37,38,39,40,41,42]. This in vitro model encompasses the main stages of myogenesis, especially myoblast proliferation, differentiation, and fusion into myotubes [38,39,41]. Moreover, the cell culture system provides a more controlled environment than in vivo, allowing the analysis of many signaling pathways and molecular networks under controlled conditions. This enables a more in-depth study of regulatory molecules and the investigation of their roles at different stages of cell culture [7]. Furthermore, myoblast cell culture medium can be modified to assess the role of nutrients, growth factors or hormones in regulating the muscle growth process [12,13,14,35,38,40,42,43,44,45], such as the amino acids and IGF1.

In this context, large-scale sequencing techniques have provided enormous progress in the molecular biology field. Global approaches, such as the transcriptome and microRNAome, allow one to obtain a molecular profile of different tissues under distinct conditions or moments, providing opportunities for the identification of molecular markers, and new information on the signaling pathways that regulate a particular biological process [46]. However, to our knowledge, no study has performed large-scale sequencing in fish myotube cell cultures treated with pro-growth signals. Thus, our main objective was to obtain and evaluate the transcriptome and microRNAome of pacu (*Piaractus mesopotamicus*) myotubes stimulated with amino acids or IGF1. Our analysis of both omics provided new insight into different signaling pathways’ activation, potential molecular markers, and networks integrating gene and miRNA transcription, allowing better comprehension of the molecular regulation of fish myogenesis and muscle growth using pro-growth inputs.

## 2. Results

### 2.1. AA and IGF1 Treatments Were Effective, and AA Induced Higher Number of Differentially Expressed Genes (DEGs)

The sequencing of pacu myotubes transcriptome yielded a total of 70,363,951 (CTR), 69,999,996 (AA) and 76,376,683 (IGF1) paired-end reads per group (Supplementary Table S1). After trimming, 30,902,924 paired-end reads were successfully assembled into 176,103 contigs. A total of 69,932 contigs (40%) were successfully annotated, and after normalization, the myotubes treated with AA or IGF1 showed a non-redundant list of 1228 (524 down- and 704 up-regulated) and 534 (289 down- and 245 up-regulated) DEGs compared to the CTR group, respectively (Supplementary Tables S1 and S2).

**Figure 1:**
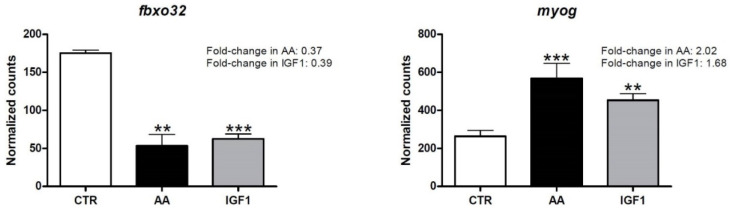
Digital expression of *fbxo32* and *myog*. Gene expression of *fbxo32* (*f-box protein 32*) and *myog* (*myogenin*) in CTR, AA and IGF1 groups according to the differential expression analyses. Expression is shown as number of counts, and values represent means ± s.e.m. (*n* = 3 independent cell cultures). The fold-changes are shown in the graphs and asterisks indicate significant differences compared to CTR: **: *p*-adj < 0.01; ***: *p*-adj < 0.001.

The principal component analysis (PCA) plot, according to the transcriptome results, showed different profiles between CTR, AA, and IGF1 samples. The pacu myotubes were well separated according to the experimental groups, showing that the treatments were effective, and the cell culture replicates were similar to each other (Supplementary Figure S1). We further confirmed the effectiveness of our protocol via the digital expression of *fbxo32* (*f-box protein 32*), a well-known marker of muscle protein degradation and atrophy [42,47,48,49,50], and *myog* (*myogenin*), related to myogenesis and growth [51,52,53,54]. Both AA and IGF1 groups showed decreased *fbxo32* transcription (fold-change = 0.37 in AA and 0.39 in IGF1; *p*-adj < 0.01) and increased *myog* transcription (fold-change = 2.02 in AA and 1.68 in IGF1; *p*-adj < 0.01) compared to CTR (Figure 1).

### 2.2. DEGs Were Specific to Each Treatment or Shared by Both AA and IGF1, Which Modulated Different Biological Processes

**Figure 2:**
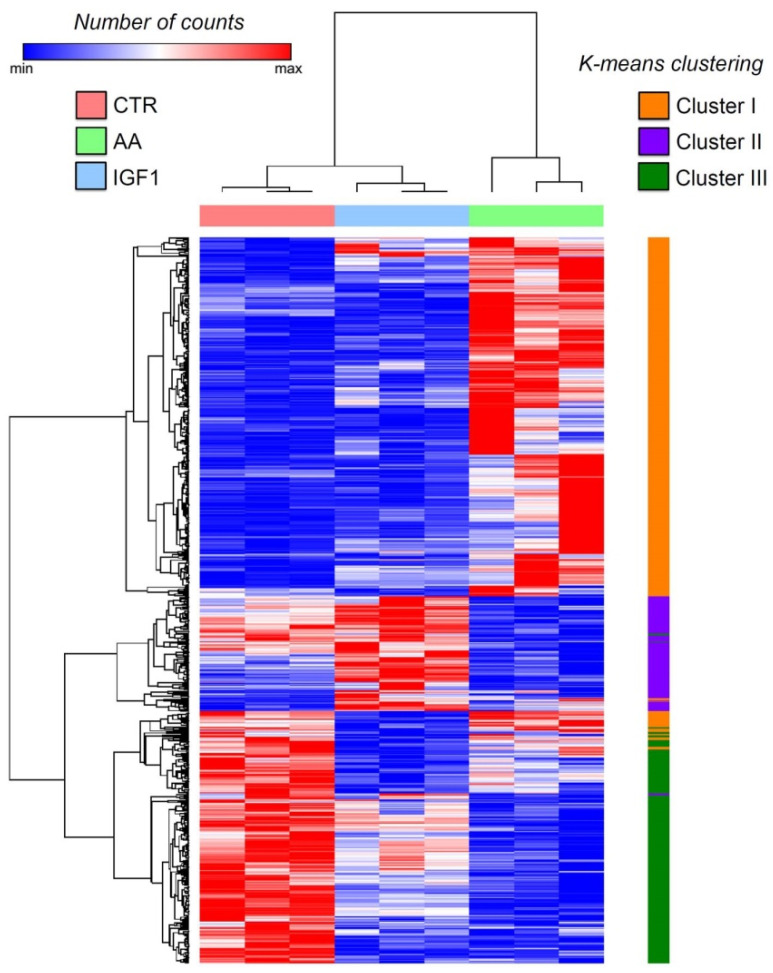
Transcriptome heatmap of pacu-cultured myotubes in CTR, AA, and IGF1 experimental groups. Heatmap showing gene expression according to the CTR, AA and IGF1 treatments by hierarchical clustering and non-hierarchical K-means clustering (K-means = 3). Heatmap shows the normalized read counts of differentially expressed genes, and one minus Pearson correlation was used as a metric for clustering.

**Figure 3:**
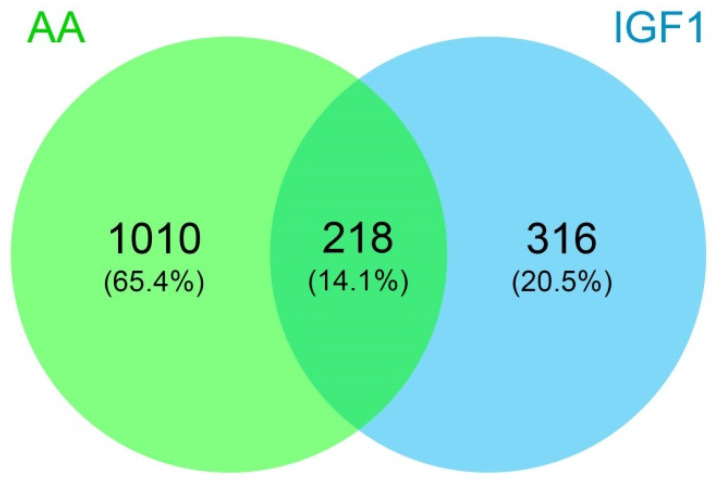
Venn diagram of genes differentially expressed in AA and IGF1 pacu-cultured myotubes. The Venn diagram showing different sets of differentially expressed genes considering the AA and IGF1 treatments, with 218 deregulated genes by both experimental groups.

The heatmap of gene expression showed a different transcription pattern between the experimental groups. The hierarchical clustering revealed three main groups of genes, better defined by the K-means clustering (K-means = 3), namely Cluster I, II and III, which have increased expression respectively in AA, IGF1, and CTR treatments (Figure 2; Supplementary Table S3). In addition, we used the Venn diagram for further information about genes up- or down-regulated by both pro-growth inputs. Among the 1228 and 534 DEGs, we found that 218 genes were differentially expressed by both AA and IGF1 treatments (Figure 3; Supplementary Table S3), mainly involved with IGF/PI3K/MTOR signaling and the JAK-STAT cascade (Supplementary Figure S2).

**Figure 4:**
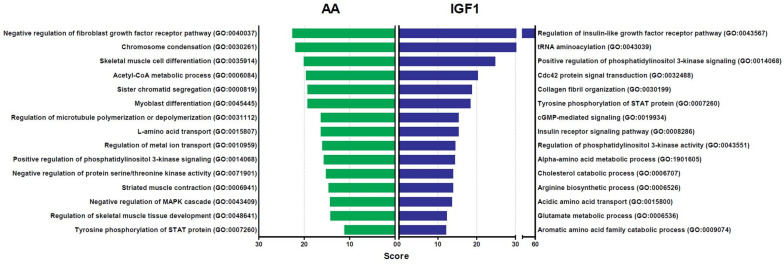
Gene ontology enrichment analysis of genes differently expressed in AA and IGF1 pacu-cultured myotubes. Biological processes were identified for up- and down-regulated genes in AA and IGF1 treatments compared to CTR group. Enrichment was defined as the 15 most significant terms according to the highest scores and *p*-values (<0.05).

A gene ontology enrichment analysis was performed to access the biological processes enriched by the DEGs in AA and IGF1-treated samples compared to the CTR group (Figure 4). Our results show that AA treatment induced the enrichment of many processes related to chromosomal alteration/mitosis (GO:0030261; GO:0000819; GO:0031112) and muscle differentiation (GO:0035914; GO:0045445; GO:0006941), while IGF1 modulated mostly IGF/PI3K signaling (GO:0043567; GO:0014068; GO:0008286), amino acids metabolism (GO:1901605; GO:0006526; GO:0015800), and matrix organization (GO:0030199) (Figure 4).

### 2.3. AA and IGF1 Treatments Resulted in Differential Expressed miRNAs, and AA Induced Higher Number of Muscle-Specific miRNAs

The sequencing of pacu myotubes microRNAome yielded a total of 40,147,542 (CTR), 40,557,507 (AA) and 44,512,081 (IGF1) single-end reads per group (Supplementary Table S1). After trimming, 122,586,164 single-end reads (98% of the total) were successfully annotated into 3579 miRNA, and after normalization, the myotubes treated with AA or IGF1 showed, respectively, 11 and 8 differentially expressed miRNA compared to the CTR group (Supplementary Tables S1 and S4).

According to the microRNAome PCA plot, the AA pacu myotubes were grouped separately from CTR and IGF1 treatments, showing that the AA input had a more distinct effect on miRNA modulation, while the samples from CTR and IGF1 groups were similar to each other (Supplementary Figure S3). In fact, the different expression analysis showed that the muscle-specific miRNAs (*miR-1*, *-133*, *-206* and *-499*) were mainly up-regulated by AA treatment, with the presence of mature sequences derived from 3p or 5p strands. In addition, our results show different paralogous copies of *miR-133* (a, b and c) up-regulated by the AA treatment (Supplementary Table S4).

### 2.4. Omics Integration Showed Complex Molecular Networks, with High marcksb/miR-29b Interaction in AA and mmp14b/miR-338-5p Interaction in IGF1

**Figure 5:**
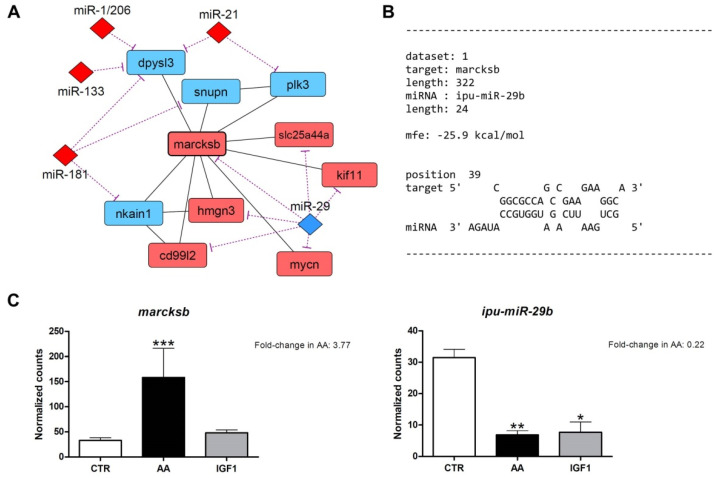
Identification of *marcksb* as potential target of *miR-29b* in AA pacu-cultured myotubes. (**A**) Interaction molecular network between *marcksb* (*myristoylated alanine rich protein kinase c substrate b*), *miR-29* and other differentially expressed genes and miRNAs. Up- and down-regulated genes are represented respectively by light red and light blue colors, and up- and down-regulated miRNAs are represented respectively by red and blue colors. Purple lines show interaction between miRNAs and genes, and black lines show interaction between the genes. (**B**) Bioinformatics prediction of the *marcksb*/*miR-29b* hybridization. The MFE (minimum free energy) value was within accepted range. (**C**) Gene expression of *marcksb* and *ipu-miR-29b* in CTR, AA and IGF1 groups according to the differential expression analyses. Expression is shown, as number of counts and values represents means ± s.e.m. (*n* = 3 independent cell cultures). The fold-changes in AA group are shown in the graphs, and asterisks indicate significant differences between groups: *: *p*-adj < 0.05; **: *p*-adj < 0.01; ***: *p*-adj < 0.001.

**Figure 6:**
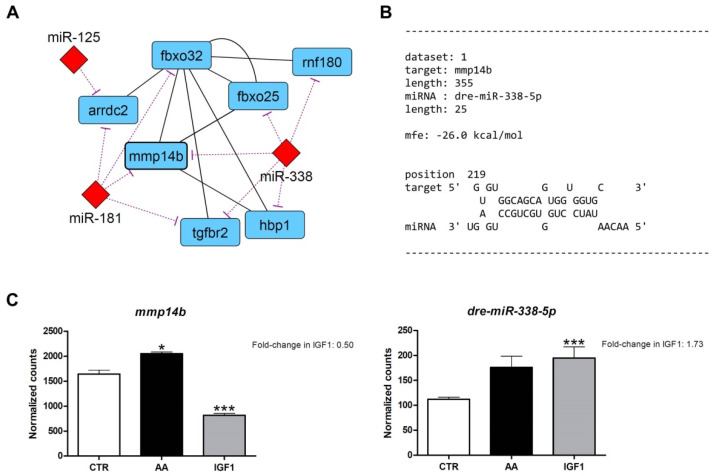
Identification of *mmp14b* as potential target of *miR-338-5p* in IGF1 pacu-cultured myotubes. (**A**) Interaction molecular network between *mmp14b* (*matrix metallopeptidase 14b*), *miR-338* and other differentially expressed genes and miRNAs. Down-regulated genes are represented by light blue color, and up-regulated miRNAs are represented by red color. Purple lines show interaction between miRNAs and genes, and black lines show interaction between the genes. (**B**) Bioinformatics prediction of the *mmp14b*/*miR-338-5p* hybridization. The MFE (minimum free energy) value was within accepted range. (**C**) Gene expression of *mmp14b* and *dre-miR-338-5p* in CTR, AA and IGF1 groups according to the differential expression analyses. Expression is shown as number of counts, and values represent means ± s.e.m. (*n* = 3 independent cell cultures). The fold-changes in IGF1 group are shown in the graphs, and asterisks indicate significant differences between groups: *: *p*-adj < 0.05; ***: *p*-adj < 0.001.

After miRNA target prediction, we found interaction molecular networks with strong relationships between the differentially expressed genes from the transcriptome, with differentially expressed miRNAs from the microRNAome. These interaction networks show several up- and down-regulated genes, co-expressed according to the literature, with potential binding sites for miRNAs altered by AA or IGF1 treatments (Supplementary Figure S4). Within the networks generated for AA treatment, we found *marcksb*/*miR-29b* interaction with high hybridization (MFE = −25.9 kcal/mol). Moreover, MARCKS is involved in myogenesis and muscle differentiation [55,56,57,58,59,60], and showed a number of interactions with others DEG in transcriptome (Figure 5). In the IGF1 network, we found *mmp14b*/*miR-338-5p* interactions with high hybridization (MFE = −26 kcal/mol). MMP14 also showed interactions with other DEGs in the transcriptome, besides the involvement in muscle regeneration and fibrous tissue organization and development [61,62,63,64] (Figure 6). In addition, to complement our results and provide further insight, we used the counts obtained in our work to check the correlation between miRNA–target interactions already validated in the literature (Supplementary Figure S5).

### 2.5. Inverse Expression Pattern Was Observed between marcksb and miR-29b, and between mmp14b and miR-338-5p, Both In Vitro and In Vivo

**Figure 7:**
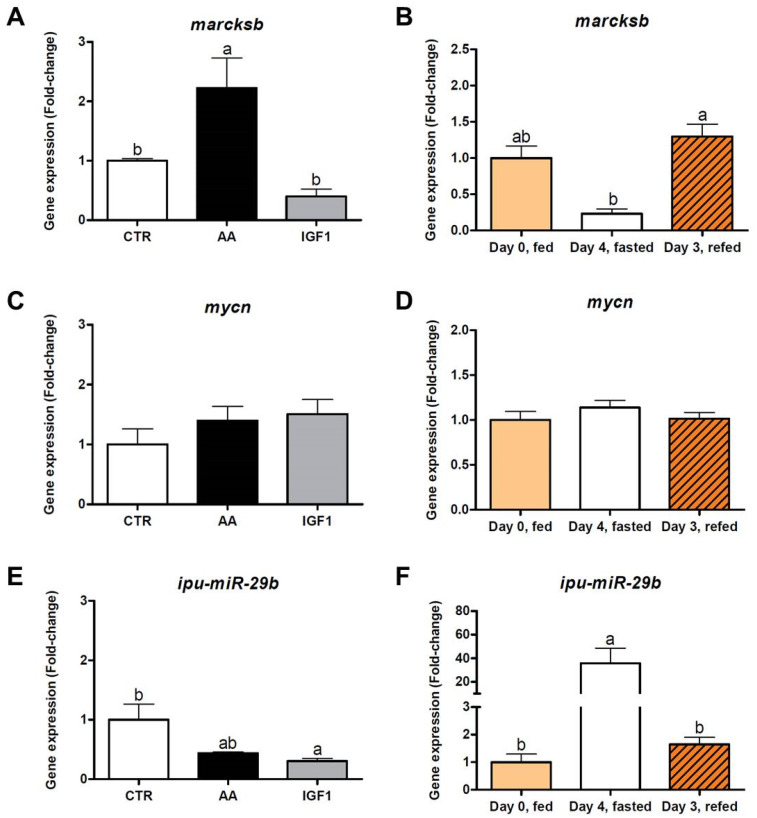
(**A**,**C**,**E**) In vitro and (**B**,**D**,**F**) in vivo relative expression of *marcksb*, *mycn* and *miR-29b*. Relative gene expression of *marcksb* (*myristoylated alanine rich protein kinase C substrate b*), *mycn* (*n-myc proto-oncogene protein*) and *ipu-miR-29b* by qPCR. Validation was performed from CTR, AA, and IGF1 myotubes (in vitro samples; *n* = 4 independent cell cultures), and from fish muscles before fasting (Day 0, fed), after 4 days of fasting (Day 4, fasted), and 3 days of re-feeding (Day 3, refed) (in vivo samples; *n* = 6). Values represent means ± s.e.m. Letters indicate significant differences between groups. Parametric data were analyzed by one-way ANOVA test, followed by Tukey’s multiple comparisons test, while non-parametric data were analyzed by a Kruskal–Wallis test, followed by Dunn’s multiple comparisons test (*p* < 0.05).

To validate and further explore our results, we evaluate the gene expression of *marcksb*, *miR-29b*, *mmp14b*, *miR-338-5p*, and other genes selected from AA and IGF1 networks (Supplementary Table S5). We used in vitro pacu myotubes treated with amino acids or IGF1, and in vivo muscles from pacus submitted to fasting (4 days) and re-feeding (3 days). The *marcksb* transcription was increased in myotubes treated with AA (fold-change = 2.23; *p* < 0.05) compared to CTR and IGF1, and increased in the re-fed fish (fold-change = 1.30; *p* < 0.01) compared to the fasting period. The expression of *mycn* did not show statistical differences between experimental groups. On the other hand, *miR-29b* expression was decreased in AA, despite the lack of statistical differences, and in IGF1, compared to CTR (fold-change = 0.30; *p* < 0.05). Similarly, *miR-29b* showed up-regulation after fasting (fold-change = 35.7; *p* < 0.05) and down-regulation after refeeding (fold-change = 1.64; *p* < 0.05) (Figure 7).

**Figure 8:**
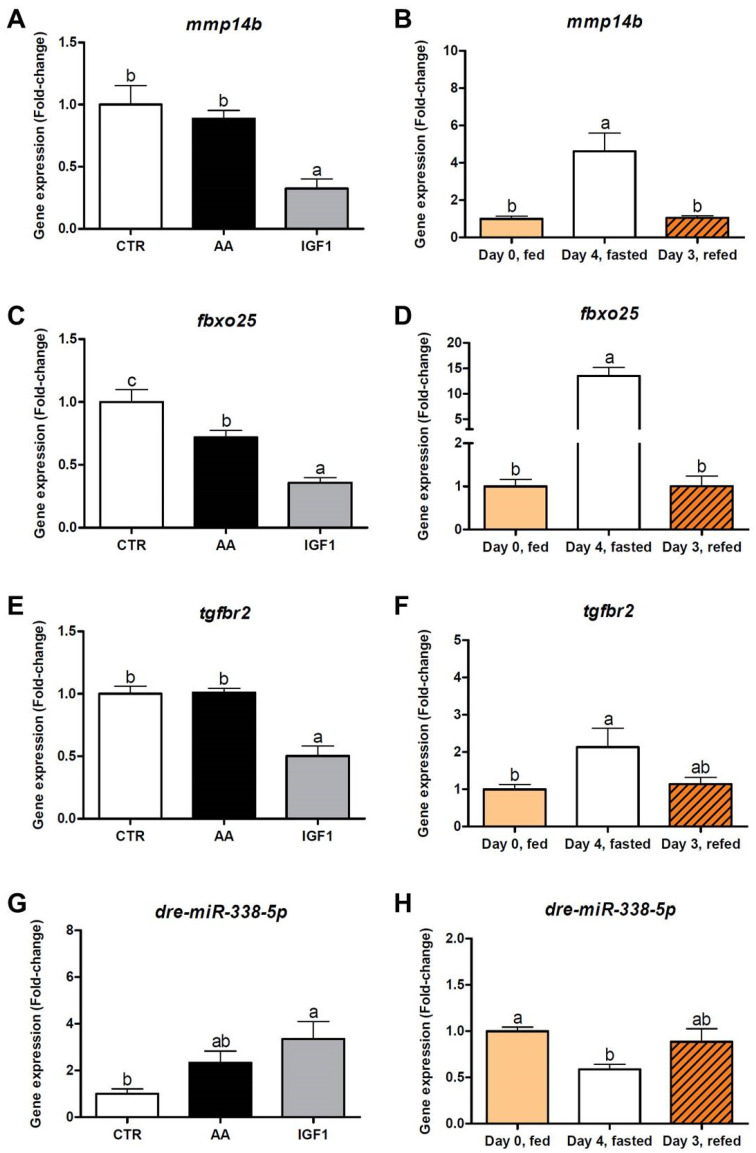
(**A**,**C**,**E**,**G**) In vitro and (**B**,**D**,**F**,**H**) in vivo relative expression of *mmp14b*, *fbxo25*, *tgfbr2* and *miR-338-5p*. Relative gene expression of *mmp14b* (*matrix metallopeptidase 14b*), *fbxo25* (*f-box protein 25*), *tgfbr2* (*tgf-beta receptor type-2*) and *dre-miR-338-5p* by qPCR. Validation was performed from CTR, AA and IGF1 myotubes (in vitro samples; *n* = 4 independent cell cultures), and from fish muscles before fasting (Day 0, fed), after 4 days of fasting (Day 4, fasted), and 3 days of re-feeding (Day 3, refed) (in vivo samples; *n* = 6). Values represent means ± s.e.m. Letters indicate significant differences between groups. Parametric data was analyzed by one-way ANOVA test, followed by Tukey’s multiple comparisons test, while non-parametric data were analyzed by the Kruskal–Wallis test, followed by Dunn’s multiple comparisons test (*p* < 0.05).

The expression of *mmp14b*, *fbxo25* and *tgfbr2* was decreased in myotubes treated with IGF1 compared to other groups (fold-change = 0.32 for *mmp14b*, fold-change = 0.36 for *fbxo25* and fold-change = 0.50 for *tgfbr2*; *p* < 0.01), results corroborated by the increased transcription of these genes after fasting (fold-change = 4.61 for *mmp14b*, fold-change = 13.5 for *fbxo25* and fold-change = 2.13 for *tgfbr2*; *p* < 0.05), and decreased transcription after re-feeding (fold-change = 1.04 for *mmp14b* and fold-change = 1.00 for *fbxo25*; *p* < 0.01). In contrast, the *miR-338-5p* showed up-regulation in IGF1 compared to CTR (fold-change = 3.35; *p* < 0.05), while the fish showed decreased transcription after fasting (fold-change = 0.59; *p* < 0.05), and a slight increase after re-feeding, despite the lack of statistical difference (Figure 8).

In addition, to complement our results, we checked the correlation between *marcksb*/*miR-29b* and *mmp14b*/*miR-338-5p* interactions. Both showed significant negative correlation indexes (ρ = −0.35 and ρ = −0.62, respectively; *p* < 0.05) (Supplementary Figure S6).

## 3. Discussion

Research on fish skeletal muscle growth has enormous importance in fish farming production and development, with this tissue representing the main product of the aquaculture industry [1,7]. Hyperplasic and/or hypertrophic muscle growth involve the proliferation and differentiation of myoblasts and their subsequent fusion into myotubes, steps recapitulated by the fish myoblast cell cultures [38,39,41]. In addition, fish muscle growth is influenced by different inputs that lead to increased protein synthesis, strongly regulated by the IGF/PI3K/MTOR axis. Among them, the IGF1 itself and several amino acids are the most studied factors that promote muscle growth [41].

Different studies have used the fish myoblast cell cultures to examine anabolic pathways in muscle [11,12,13,44,65]. Díaz et al. (2009) detected *glut4* up-regulation (*insulin-responsive glucose transporter type 4*) in both myoblasts and myotubes after treatment with INS (insulin) and IGF1 [66]. Moreover, IGF1 and INS supplementation to trout myocytes promoted increased protein synthesis and decreased proteolysis, whereas the administration of leucine only reduced protein degradation [44]. In addition, the lack of amino acids increases autophagosome formation, the expression of autophagy genes [67], and the transcription of *fbxo32*/*mafbx* and *murf1* (*muscle-specific ring finger protein 1*) [68], but there is evidence that amino acids can act as positive or negative regulators of protein turnover in fish muscle. Cleveland and Radler (2019) showed that leucine and phenylalanine directly regulate proteolysis in rainbow trout-cultured muscle cells, with leucine as a central regulator of protein turnover, while an excess of lysine and valine increased rates of protein degradation [69]. On the other hand, Azizi et al. (2016) observed that lysine deficiency down-regulated the expression of IGF signaling components and MRFs in gilthead sea bream myocytes [70].

In our study, we obtained a higher amount of DEGs in AA-treated myotubes (1228), with much fewer de-regulated genes in IGF1-treated cells (534) (Supplementary Tables S1 and S2), which could be an indicative that AA treatment was more effective or potent than the pro-growth condition, compared to the IGF1 group. However, we must consider that the strong regulation of AA treatment may result from the medium composition, which includes several amino acids with unique or overlapping roles in muscle growth and metabolism [69], representing a collection of anabolic factors, and not a single factor as the IGF1. According to the heatmap, there is a gene expression signature specific to each experimental group, CTR (Cluster III), AA (Cluster I), or IGF1 group (Cluster II) (Figure 2). Considering the distinct molecular repertories that are mobilized in each pro-growth condition, the identification of the genes in each cluster (Supplementary Table S3) could be of interest for research focusing on AA or IGF1 treatment. Our results provided important large-scale data from isolated fish muscle cells treated with the most used inputs to increase myogenesis and muscle growth [41], something not yet investigated, to the best of our knowledge. In addition, we identified 218 genes with up- or down-regulated transcription in both AA and IGF1 treatments (Figure 3; Supplementary Table S3). Among these genes, we found the down-regulation of *igfbp1a* (*insulin-like growth factor-binding protein 1*; fold-change = 0.31 in AA and 0.35 in IGF1) and up-regulation of *igfbp5a* (insulin-like growth factor-binding protein 5; fold-change = 2.34 in AA and 2.16 in IGF1), both with defined roles in skeletal muscle.

Despite having structural similarities, IGFBPs exert different functions according to the physiological conditions and cell types [71,72,73]. In teleost fish, as well as in mammals, the highest expression of *igfbp1* occurs in the liver under normal conditions [74,75], and in skeletal muscle under low nutrient availability. To overcome this catabolic condition, IGFBP1 possibly sequesters IGFs from IGFRs, prioritizing metabolic processes associated with cell survival instead of muscle growth [72]. In this sense, Rolland et al. (2015) described that the low amino acid diet promotes the up-regulation of *igfbp1* in rainbow trout, a process reversed when the anabolic environment is recovered [76]. Similarly, the down-regulation of *igfbp1a* in both the AA and IGF1 treatments of our study indicates the activation of anabolic pathways and the continuity of myogenic processes. On the other hand, studies have shown that *igfbp5* has great importance in myogenesis, muscle growth and gill functions, regulating IGFs and calcium ion influx [77,78,79]. Atlantic salmon muscle cells had high expression of *igfbp5* paralogs (*igfbp5a* and *igfbp5b*) in response to amino acids, possibly stimulating the cell cycle [13], and gilthead sea bream muscle cells showed increased *igfbp5* transcription under treatment with amino acids and IGFs [70,80], similar to our data, results that corroborate and reinforce the importance of this gene in pro-growth conditions.

Moreover, our results also showed that individual AA and IGF1 treatments modulated different signaling pathways. AA samples showed the enrichment of biological processes related to chromosomal changes/mitosis and muscle differentiation, while IGF1 samples induced IGF/PI3K signaling, the metabolism of amino acids, and matrix organization (Figure 4). These same biological processes were enriched in each treatment considering the exclusive genes in heatmap clusters. Corroborating our data, studies showed that AA stimulates protein synthesis during myogenesis by themselves [11,12], through the direct activation of the MTOR complex, without affecting upstream components such as PI3K, but IGF1 only stimulates protein synthesis if AA are also present [13,14]. In fact, our results indicate that PI3K signaling is less enriched in AA myotubes, and that IGF1 requires the activation of amino acids metabolism to stimulate protein synthesis. It seems that IGF1 has an upstream effect to induce muscle growth, in accordance with its role as a binding factor at the muscle cell membrane [81], stimulating protein synthesis via an increase in PI3K signaling. On the other hand, the AA treatment appears to have a downstream effect on muscle growth, increasing myogenesis through cell proliferation/mitosis and differentiation. It is not clear why IGF1 alone fails to stimulate myogenic pathways such as AA. A hypothesis would be that amino acids are more relevant and consistent during the MTOR integration of nutritional and hormonal signals to regulate protein synthesis and cell proliferation [82,83,84]. The amino acids may facilitate IGF1 function, and their absence in IGF1 treatment possibly inhibited, or did not activate, components of the IGF and myogenic systems, similar to the results of Azizi et al. (2016) [70]. However, as discussed, this broader effect of AA treatment may result from the presence of several different amino acids in the culture medium.

The miRNAs also regulate all steps of myogenesis [29,30,31,32,85,86], through the silencing of mRNAs [16,17,18]. In our study, we observed, respectively, 11 and 8 differentially expressed miRNAs in AA and IGF1 samples (Supplementary Tables S1 and S4), with the majority of the AA up-regulating muscle-specific miRNAs (*miR-1*, *-133*, *-206* and *-499*), which could also indicate the higher potency of AA treatment as a pro-growth input. Both in mammals and teleost fish, the *miR-1* and *-206* belong to the same miRNA family and are involved in muscle development by stimulating myoblast differentiation [29,30,34], while the myoblast proliferation is regulated by *miR-133* [29,34]. On the other hand, *miR-499* participates in the specification and maintenance of the slow-twitch muscle fiber phenotype, with increased expression in slow muscle cells [31,32,34,36,87,88]. Our work showed significant negative correlation between miRNAs and validated targets involved with myogenesis and muscle growth (Supplementary Figure S5), and the high enrichment of myogenic processes in AA myotubes could be explained by the up-regulation of several muscle-specific miRNAs. The favored cell differentiation was stimulated by *miR-1* and *miR-206* (fold-change = 1.25 and 2.39 in AA), while myoblast proliferation/mitosis was probably regulated by the many paralogous copies of *miR-133* (fold-change = 1.75 in AA), which also showed different active strands (5p and 3p) (Supplementary Table S4). The different copies of *miR-133* (*-133a*, *-133b* and *-133c*) and their specific functions in muscle growth constitute an interesting research field, leading to improvements in biological knowledge, and the potential to apply this in aquaculture.

Rather than individual genes and miRNAs, both AA and IGF1 treatments also activated large sets of molecular components, demonstrated by the strong relationship and high complexity of the interaction networks (Supplementary Figure S4). Within such networks, we found two interesting genes: *marcksb* (fold-change = 3.77 in AA), which is related to myogenesis and muscle differentiation; and *mmp14b* (fold-change = 0.5 in IGF1), which is related to matrix organization and development (Figure 5 and Figure 6). These de-regulated genes showed high affinity with the miRNAs *miR-29b* (fold-change = 0.22 in AA) and *miR-338-5p* (fold-change = 1.73 in IGF1), respectively. Validation through qPCR corroborated the results of differential gene expression, with increased *marcksb* expression and decreased *mmp14b* expression by pro-growth stimuli both in vitro and in vivo (Figure 7 and Figure 8), besides a significant negative correlation between these genes and miRNAs (Supplementary Figure S6).

The MARCKS is an actin-binding protein that translocates from plasma membrane to cytosol and vice versa, depending on its phosphorylation state, which is regulated mainly by protein kinase C [55,60]. Studies showed that MARCKS translocation regulates muscle cell adhesion, spreading [56], differentiation and fusion [57,58], in addition to the control of cytoskeleton dynamics [89]. Moreover, the blocking of MARCKS resulted in abnormalities in the skeletal muscle of zebrafish, with an increased number of nuclei and curve-shaped fibers [59]. Our work showed that AA treatment stimulated the enrichment of muscle differentiation processes, consistent with the up-regulation of *marcksb* by AA in in silico (Figure 5A,C), in vitro (Figure 7A), and in vivo re-fed pacus (Figure 7B). Interestingly, other well-characterized genes of myoblast fusion were also up-regulated by the AA in our in silico analyses, such as *myogenin* (fold-change = 2.02) and *myomaker* (fold-change = 2.12), indicating that *marcksb* could be a new option and a possible molecular marker of fish muscle cell differentiation in pro-growth conditions. In contrast, *miR-29b* was down-regulated by both AA and IGF1 in in silico (Figure 5A,C), in vitro (Figure 7E) and in vivo re-fed pacus (Figure 7F). In fact, *miR-29b* is commonly up-regulated in multiple types of muscle atrophy, as induced by denervation, dexamethasone, fasting, ageing or cachexia, conditions attenuated after the inhibition of this miRNA [90,91]. The reduced expression of *miR-29b* in our AA treatment probably allowed the effects of *marcksb,* and favored the environment for myogenesis and muscle growth. To obtain further information, we also evaluated the expression of *mycn*, a proto-oncogene required for cell proliferation which inhibits myogenic differentiation [92]. Despite the up-regulation of *mycn* in AA transcriptome (fold-change = 2.56) and the connection with *marcksb* in the molecular network (Figure 5A), we did not find statistical differences between the groups, in vitro or in vivo (Figure 7C,D), which reinforces a more active role of *marcksb* and enhanced muscle differentiation.

MMP14 is a collagenase and a member of the matrix metalloproteinase family, which is not fully characterized in skeletal muscle. Ohtake et al. (2006) demonstrated that MMP14 is a major contributor to the progression of myogenesis through the degradation of extracellular matrix (ECM) components. In addition, MMP14-deficient mice showed smaller and more heterogeneous muscle fibers with compromised integrity [62]. Human muscle satellite cells also showed the expression of this metalloproteinase, which is necessary for in vitro cellular invasion through collagen I [61], highlighting the relevance of MMP14 activity for muscle cell migration and ECM remodeling, which are particularly important for muscle regeneration and growth [63]. In our study, we observed the down-regulation of *mmp14b* by IGF1 in silico (Figure 6A,C), in vitro (Figure 8A) and in vivo re-fed pacus (Figure 8B). Although controversial, these results make sense considering that IGF1 mainly enriched processes related to IGF/PI3K signaling, and not myogenic mechanisms. In fact, *mmp14b* was up-regulated in the AA transcriptome (Figure 6C). As discussed, IGF1 treatment was possibly less effective as a pro-growth condition compared to the AA group, suggesting that the cells were focusing on protein turnover rather than muscle proliferation and differentiation. On the other hand, *miR-338-5p* was up-regulated by IGF1 in in silico (Figure 5A,C), in vitro (Figure 8G) and in vivo re-fed pacus (Figure 8H). To the best of our knowledge, no study has evaluated this miRNA in fish skeletal muscle. Lei et al. (2017) have shown roles of *miR-338-5p* in suppressing the proliferation and migration of glioblastoma cells through the inhibition of EFEMP1, a regulator of matrix metalloproteinases [93], and Nielsen et al. (2014) observed an up-regulation of *miR-338-3p* in circulation after 1 h of acute exercise [94], allowing us to draw a parallel with the pro-growth effect of IGF1.

For a deeper understanding of our in silico results, we also evaluated the expression of other de-regulated genes that showed an interaction with *mmp14b* and could also be regulated by *miR-338-5p*. In association with *mmp14b*, we found the decreased expression of *fbxo25* and *tgfbr2* after the pro-growth stimuli, both in vitro and in vivo (Figure 8C–F). The FBXO25 is classified as an E3 ubiquitin ligase with high homology to FBXO32 in fish [95]. We previously demonstrated the increased expression of *fbxo25* during fasting and down-regulation during re-feeding in pacu muscle and cultured cells [42,96], and this same molecular scenario was observed in fasted and re-fed rainbow trout [97]. In the present work, a pronounced reduction in *fbxo25* expression was observed in IGF1, indicating that the protein breakdown should be stopped, so that anabolism and muscle growth take place in the treatment. The TGFB pathway and its downstream components are well known as myogenic inhibitors, with MSTN (myostatin) widely studied in fish muscle development [98]. The TGFB signaling starts with the binding of the ligand to type 1 and 2 membrane receptors, forcing their assembly into a complex that initiates the phosphorylation cascade [99]. In this sense, the activation of *tgfbr2* was associated with *myod* and *myog* down-regulation in C2C12 cells, and the *tgfbr2* inhibition by miRNA resulted in a reversed effect [100]. In addition, Accornero et al. (2014) showed that the blocking of TGFB signaling through TGFBR2 mutant attenuated muscular dystrophy and injury, and improved muscle regeneration and satellite cell numbers in mice [101], indicating a negative regulation by *tgfbr2* in muscle growth. In the present work, the down-regulation of *tgfbr2*, both in vitro and in vivo, demonstrates that the suppression of TGFB signaling may be necessary for fish muscle growth regulated by IGF1. Our results suggest that IGF1-induced the expression of *miR-338-5p*, which possibly decreased the expression of *mmp14b*, *fbxo25* and *tgfbr2*, resulting in positive effects for protein synthesis and anabolism, but supressing the progression of myogenesis to avoid excessive cell energy expenditure.

## 4. Materials and Methods

### 4.1. Fish and Sample Collection

All experiments and procedures were performed in accordance with the Ethical Principles in Animal Research adopted by the National Council for the Control of Animal Experimentation (CONCEA). The protocol was approved by the Ethics Committee on Animal Use of the Institute of Biosciences, São Paulo State University (UNESP), Botucatu (protocol number 1184; 14 June 2019) and of the Federal University of Goiás (UFG), Goiânia (protocol number MB 026/21; 20 April 2021). The experiments were also conducted following the ARRIVE guidelines [102].

Fish were farmed at 28 °C under 12 h light:12 h dark photoperiod in storage tanks of 0.5 m^3^ equipped with water circulation system. For in vitro experiments, juvenile pacus (5–20 g, *n* = 20 per culture) were fed ad libitum once a day with a commercial diet, remaining 24 h in fasting before the experiments. For in vivo experiments, juvenile pacus (10–15 g, *n* = 6 per group) were submitted to fasting/re-feeding protocol, with a commercial diet. Fast-twitch muscles were collected from the epaxial region before the fasting protocol (Day 0), after 4 days of fasting (Day 4), and after 3 days of re-feeding (Day 3). All fish were euthanized with an excess of benzocaine (≥250 mg/L; Sigma-Aldrich, St. Louis, MO, USA) prior to body weight (g) measuring and muscle collection.

### 4.2. Isolation and Myoblast Cell Cultures

The myoblasts were isolated and cultured according to the protocol described by Fauconneau and Paboeuf (2000) [103]. The fast-twitch muscles were collected from the epaxial region and mechanically dissociated with scalpels. To release the muscle cells, the fragments were enzymatically digested with 0.2% collagenase type I (C9891) and 0.1% trypsin (T4799) (Sigma-Aldrich, St. Louis, MO, USA). The cell suspension was filtered in cell strainers (Corning, New York, NY, USA), allowing for the removal of debris, centrifuged, and the cell pellet was resuspended in DMEM medium (DMEM (D7777), 9 mM NaHCO_3_ (S5761), 20 mM HEPES (H3375), pH 7.4), with 1% antibiotics (A5955) and 10% fetal bovine serum (F7524) (Sigma-Aldrich, St. Louis, MO, USA). The cells were diluted at a concentration of 2 × 10^6^ cells/mL and plated in 6-wells plates, previously treated with poly-L-lysine (P6282) and laminin (L2020) (Sigma-Aldrich, St. Louis, MO, USA), which have high affinity for the myoblasts. The myoblasts were incubated at 28 °C, with the medium changed every day, and the myoblasts morphology was monitored regularly under a microscope (Olympus, Tokyo, Japan). The results were achieved from 3 independent cell cultures.

### 4.3. Amino Acids and IGF1 Treatments

After 8 days of cell culture (myotube formation), cells were incubated for 12 h with free amino acid medium (Earle’s balance salt solution 1× (E7510), 9 mM NaHCO_3_, 20 mM HEPES, Vitamin Mix 1× (M6895), 1% antibiotics and 4 g/L D-glucose (G8270)—Sigma-Aldrich, St. Louis, MO, USA) to reduce gene expression to basal levels. The pacu myotubes were incubated for additional 24 h in free amino acid medium (CTR group), medium with amino acids (AA group) (DMEM, 9 mM NaHCO_3_, 20 mM HEPES and 1% antibiotics—Sigma-Aldrich, St. Louis, MO, USA), or medium with recombinant IGF1 (IGF1 group) (free amino acid medium supplemented with IGF1 from gilthead sea bream at 100 ng/mL—ProSpec, Rehovot, Israel). The treatments were performed according to the protocol described by Bower and Johnston (2010) and Garcia de la serrana and Johnston (2013) [13,14]. Although minor differences, the final media composition between the groups differed, essentially with respect to the presence of amino acids or IGF1.

### 4.4. RNA Extraction and Sequencing

Total RNA was extracted from the myotubes immediately after the treatments, using TRIzol^®^ Reagent (Thermo Fisher Scientific, Waltham, MA, USA), following the manufacturer’s guidelines. RNA quantification and purity were estimated by spectrophotometry using 260/280 and 260/230 ratios (NanoVue™ Plus GE Healthcare, Chicago, IL, USA), and only samples with ratios >2.0 were used. The RNA integrity was evaluated through capillary electrophoresis using the 2100 Bioanalyzer System (Agilent, Santa Clara, CA, USA), and samples with RNA integrity number (RIN) >9.5 were used.

The generation of DNA libraries and sequencing of mRNAs and miRNAs were performed by LC Sciences (Houston, TX, USA). Transcriptome was obtained through the NovaSeq 6000 platform (Illumina, San Diego, CA, USA) with 150 base pairs, paired-end, and 6 GB data per sample. microRNAome was obtained through the HiSeq 4000 platform (Illumina, San Diego, CA, USA) with 50 base pairs, single-end, and 7–10 million reads per sample. The resulting large-scale sequencing data were processed through the GNU/Linux operating system, based on the Linux Mint 18.1 distribution (www.linuxmint.com, accessed on 3 May 2020).

### 4.5. Transcriptome and microRNAome Analyses

The quality of the sequencing was evaluated through the software FastQC version 0.11.8 (www.bioinformatics.babraham.ac.uk/projects/fastqc, accessed on 3 May 2020), and the software Trimmomatic [104] was used for adapters removal and filtering of the reads by quality, using a phred score >33. Considering the mature miRNA size (~22 nucleotides), only reads more than 17 nucleotides in length were maintained for the microRNAome.

For the transcriptome, the paired-end reads were de novo assembled using the Trinity software [105,106], and the contigs were annotated using Basic Local Alignment Search Tool (BLAST) [107] and OmicsBox software (version 1.4.12, BioBam Bioinformatics; https://www.biobam.com/omicsbox, accessed on 3 May 2020). Sequences were blasted against the Ostariophysi fish proteome database (*Astyanax mexicanus*, *Danio rerio* and *Pygocentrus nattereri*) and downloaded from the Ensembl Genome Browser 89 (http://www.ensembl.org/index.html, accessed on 3 May 2020), using BLASTx with an e-value cut-off of 10^−3^. The annotated transcriptome was used to map the contigs for each sample, using the Bowtie2 aligner [108]. For the microRNAome, the Bowtie2 aligner was used for the annotation and mapping steps based on the mature miRNA list of teleost fish (*Astatotilapia burtoni*, *Cyprinus carpio*, *Danio rerio*, *Takifugu rubripes*, *Gadus morhua*, *Ictalurus punctatus*, *Metriaclima zebra*, *Neolamprologus brichardi*, *Oryzias latipes*, *Oreochromis niloticus*, *Pundamilia nyererei*, *Salmo salar* and *Tetraodon nigroviridis*) available in miRBase version 22.1 (www.mirbase.org, accessed on 3 May 2020), allowing us to obtain an expression profile of known miRNAs.

Tables with the contig counts were submitted to differential expression analyses using the Bioconductor/R software with the DESeq2 package [109,110]. Genes and miRNAs counts were normalized by the median of the ratios method, with the counts divided by sample-specific size factors. Considering the number of counts between the samples, only genes and miRNAs with counts mean ≥10 were maintained. The genes were considered differentially expressed with adjusted *p*-value ≤ 0.05 and Log_2_(Fold-change) ≤−1 and ≥1 (2-fold). The miRNAs were considered differentially expressed with adjusted *p*-value ≤ 0.05 and Log_2_(Fold-change) ≤−0.55 and ≥0.55 (1.5-fold). All the raw and processed data of transcriptome and microRNAome analyses are available in the Gene Expression Omnibus (GEO) DataSets under the accession number GSE192683.

### 4.6. Principal Component Analysis, Heatmap, Venn Diagram and Gene Ontology Enrichment Analysis

A principal component analysis (PCA) related to gene and miRNAs analysis was performed using the number of normalized counts in log2 scale in Bioconductor/R software with the DESeq2 package [109,110]. The heatmap was created using Morpheus software (https://software.broadinstitute.org/morpheus, accessed on 18 August 2020) [111] with the normalized number of counts in the transcriptome. The hierarchical clustering was performed using one minus Pearson correlation as a metric and average linkage method. Non-hierarchical K-means clustering, obtained using the same metric, was used to better define different clusters of genes among the expression data. The Venn diagram was obtained in the Venny 2.1 software (https://bioinfogp.cnb.csic.es/tools/venny/index.html, accessed on 18 August 2020) and used to show sets of DEGs appearing in one or both AA vs. CTR and IGF1 vs. CTR comparisons. A gene ontology (GO) enrichment analysis was performed using the FishEnrichr database [112,113]. Up- and down-regulated genes were used to identify over-represented gene ontology terms of biological processes based on the annotation for *Danio rerio*. The Fisher exact was used as test type and, for each comparison, we considered the 10–15 most enriched terms according to the highest scores and lowest *p*-values (≤0.05).

### 4.7. miRNA target Prediction and Interaction Molecular Networks

The prediction of target mRNAs of the differentially expressed miRNAs was performed using TargetScanFish 6.2 [114], by searching for the presence of 8mer and 7mer sites that match the seed region of each miRNA. Interaction molecular networks were generated with the Cytoscape software [115], based on the *Danio rerio* annotation, using the GeneMANIA plugin [116], in order to show the level of the relationship between genes and miRNA, and the potential altered signaling pathways.

The miRNA target predictions were further evaluated by computational approaches, using the TransDecoder version 5.5.0 software [106] to identify the open reading frame and the ExUTR version 0.1.0 [117] to extract the 3′ untranslated regions of the mRNAs’ sequences. RNAhybrid version 2.1.2 [118] was used with the default parameters to predict miRNA target interaction, providing the minimum free energy (MFE) of hybridization and potential binding sites through nucleotide base complementarity. We selected miRNA target interactions with MFE ≤ −25 kcal/mol. In addition, we used the number of counts to analyze the correlation between miRNAs and genes that are well-validated according to the literature. The correlation index and statistical significance between miRNA and their targets were estimated using a Pearson correlation test, and the graphs were constructed using the ggplot2 R package [119].

### 4.8. Genes and miRNAs Validation by qPCR

For qPCR validation, we selected genes and miRNAs from the molecular networks which showed better MFE values of hybridization. We used cultured pacu myotubes kept in free amino acid medium, treated with amino acids or IGF1, as described (in vitro samples; *n* = 4), and pacu skeletal muscles before fasting, after 4 days of fasting and 3 days of re-feeding (in vivo samples; *n* = 6). Extracted RNA was treated with DNase I, Amplification Grade (Thermo Fisher Scientific, Waltham, MA, USA) to eliminate any possible contaminating genomic DNA from the samples, and reverse-transcribed using the High-Capacity cDNA Archive Kit (Thermo Fisher Scientific, Waltham, MA, USA). For miRNA expression, each cDNA was amplified using the TaqMan^®^ Universal PCR Master Mix (Thermo Fisher Scientific, Waltham, MA, USA) and the TaqMan^®^ MicroRNA Assays (Thermo Fisher Scientific, Waltham, MA, USA), which contains primers and specific probes to *miR-29b*, *miR-338-5p* and *U6 snRNA* (*U6 small nuclear RNA*). For the *marcksb* (*myristoylated alanine rich protein kinase C substrate b*), *mycn* (*n-myc proto-oncogene protein*), *mmp14b* (*matrix metallopeptidase 14b*), *fbxo25* (*f-box protein 25*), *tgfbr2* (*tgf-beta receptor type-2*), *ppia* (*peptidylprolyl isomerase a*) and *rpl13* (*ribosomal protein l13*) mRNAs, each cDNA was amplified using the GoTaq^®^ qPCR Master Mix (Promega, Madison, WI, USA) and primers synthesized by Exxtend (Paulínia, Brazil). The primers were designed to work at 60 °C and amplify 50–200 bp regions, expanding exon–exon boundaries when possible, using Primer3 [120]. Potential hairpins, self-dimers or cross-dimers were estimated using NetPrimer software (Premier Biosoft, San Francisco, CA, USA). All qPCR were compliant with the Minimum Information for Publication of Quantitative Real Time (MIQE) guidelines [121]. The reactions were performed in duplicate, with the following conditions: 95 °C 10 min, 40 cycles at 95 °C 15 s and 65 °C 1 min, in a QuantStudio^TM^ 12K Flex Real-Time PCR System (Thermo Fisher Scientific, Waltham, MA, USA). Primer specificity was confirmed by the presence of a single-peak dissociation curve. Relative expression was estimated using the 2^−∆∆Ct^ method [122]. The *U6 snRNA*, *ppia* and *rpl13* were selected for the normalization of expression after their stability was tested through geNorm software [123]. Statistical analyses were performed using GraphPad Prism 5 Software, as well as the construction of graphs. After a Kolmogorov–Smirnov normality test, parametric data were further analyzed by a one-way ANOVA test, followed by Tukey’s multiple comparisons test, while non-parametric data were analyzed by Kruskal–Wallis test, followed by Dunn’s multiple comparisons test. In addition, we used the fold-change values (both in vitro and in vivo experiments) to analyze and confirm the correlation between *marcksb*/*miR-29b* and *mmp14b*/*miR-338-5p*. Correlation index and statistical significance between miRNA and their targets were estimated using a Pearson correlation test, and the graphs were constructed using the ggplot2 R package [119].

## 5. Conclusions

We obtained transcriptomic and microRNAomic data from pacu-cultured myotubes submitted to AA and IGF1 treatments. These pro-growth inputs modulated different sets of genes, and AA seems to be more effective, giving the higher number of differentially expressed genes and miRNAs. In addition, AA treatment enriched processes related to chromosomal alteration/cell mitosis and muscle differentiation, while IGF1 modulated upstream PI3K signaling, and needed to stimulate amino acids metabolism to induce muscle growth. The AA samples also showed the up-regulation of muscle-specific miRNAs, which appeared to be involved in myogenic events, especially *miR-1*, *-133* and *-206*.

In addition, both AA and IGF1 down-regulated *igfbp1a* and up-regulated *igfbp5a*, interesting genes, of which the transcriptions are modulated independently of the growth treatment. These genes represent potential molecular markers and excellent candidates to be evaluated in different conditions of muscle growth, including the gain and/or loss of function assays and other customized molecular techniques. Moreover, we identified networks linking *marcksb* and *miR-29b* in AA myotubes, and *mmp14b* and *miR-338-5p* in IGF1-treated cells. These genes and miRNAs were involved in fish myogenesis and muscle growth, and could influence the way in which AA and IGF1 inputs regulate muscle physiology and metabolism.

Our results allowed the better understanding and new insights of fish muscle growth regulation by important pro-growth inputs. Together, these findings may support future research and contribute to improvements in aquaculture programs, aiming to increase muscle mass, enhance growth rate, and/or better feed conversion efficiency, as well as background information for the development and advancement of in vitro meat production.

## Data Availability

The raw and processed data of transcriptome and microRNAome analyses have been deposited on the Gene Expression Omnibus (GEO) DataSets, under the accession number GSE192683. All the produced data of the other analysis are contained within this article, or in the supplementary material.

## Supplementary Materials

The following supporting information can be downloaded at: https://www.mdpi.com/article/10.3390/ijms23031180/s1.
