# Supplementary material for: Amino Acids and IGF1 Regulation of Fish Muscle Growth Revealed by Transcriptome and microRNAome Integrative Analyses of Pacu (Piaractus mesopotamicus) Myotubes
Source: Int J Mol Sci. 2022 Jan 21;23(3):1180. doi: 10.3390/ijms23031180 (PMC8835699; doi:10.3390/ijms23031180)
Supplement: Supplementary file 1 [file ijms-23-01180-s001.zip › Figure S1.pdf]

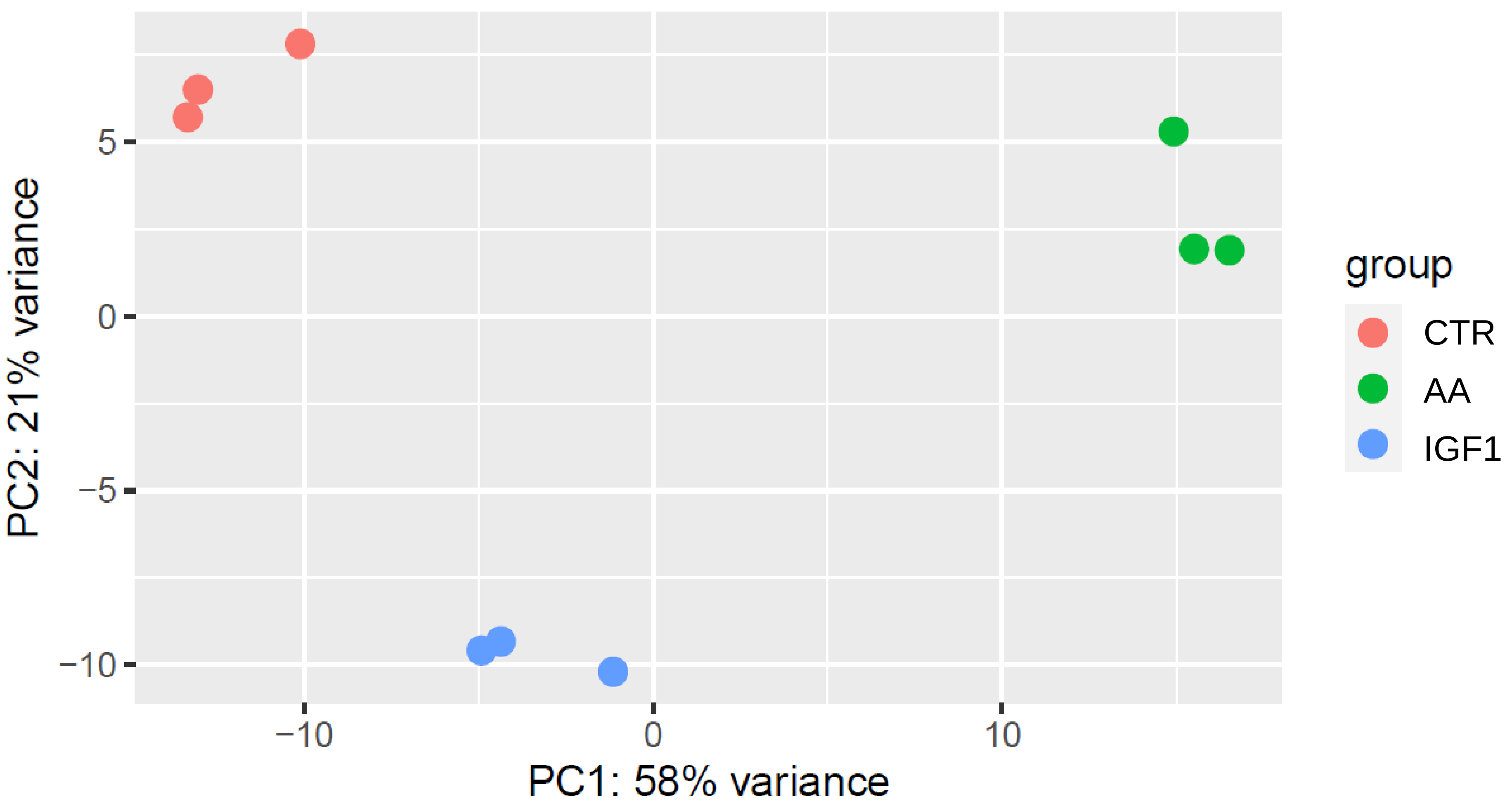

Figure S1. Transcriptome principal component analysis (PCA) of pacu cultured myotubes in CTR, AA and IGF1 experimental groups. PCA plot showing gene expression data grouped according to the CTR, AA and IGF1 treatments. Principal components were obtained using the log2 normalized counts for the genes in each sample.
