# Supplementary material for: Amino Acids and IGF1 Regulation of Fish Muscle Growth Revealed by Transcriptome and microRNAome Integrative Analyses of Pacu (Piaractus mesopotamicus) Myotubes
Source: Int J Mol Sci. 2022 Jan 21;23(3):1180. doi: 10.3390/ijms23031180 (PMC8835699; doi:10.3390/ijms23031180)
Supplement: Supplementary file 1 [file ijms-23-01180-s001.zip › Figure S2.pdf]

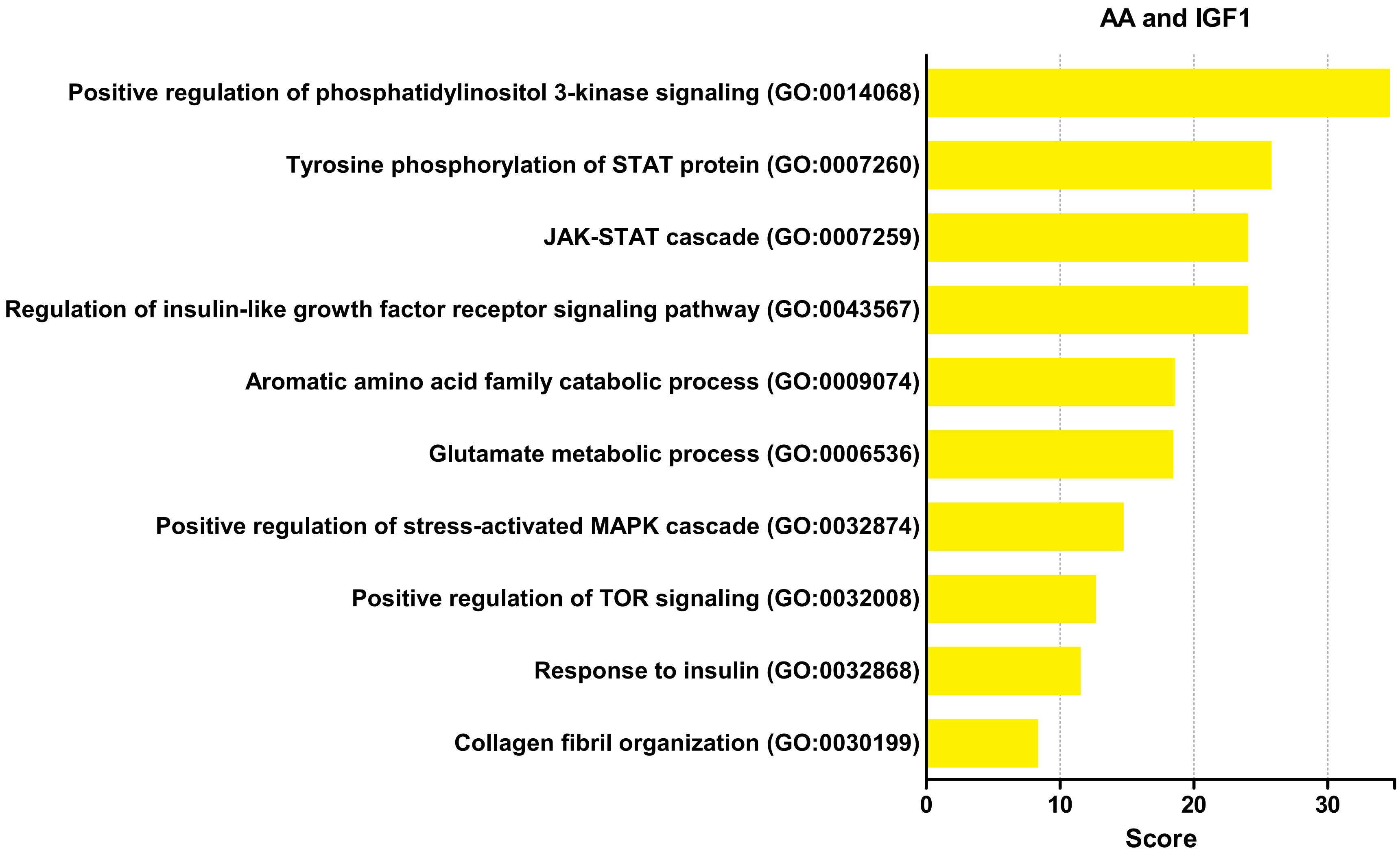

Figure S2. Gene ontology enrichment analysis of genes differently expressed in both AA and IGF1 pacu cultured myotubes. Biological processes were identified for common up- and down-regulated genes in both AA and IGF1 treatments compared to CTR group. Enrichment was defined as the 10 most significant terms according to the highest scores and P-values (<0.05).
