# Supplementary material for: Amino Acids and IGF1 Regulation of Fish Muscle Growth Revealed by Transcriptome and microRNAome Integrative Analyses of Pacu (Piaractus mesopotamicus) Myotubes
Source: Int J Mol Sci. 2022 Jan 21;23(3):1180. doi: 10.3390/ijms23031180 (PMC8835699; doi:10.3390/ijms23031180)
Supplement: Supplementary file 1 [file ijms-23-01180-s001.zip › Figure S4.pdf]

AA treatment

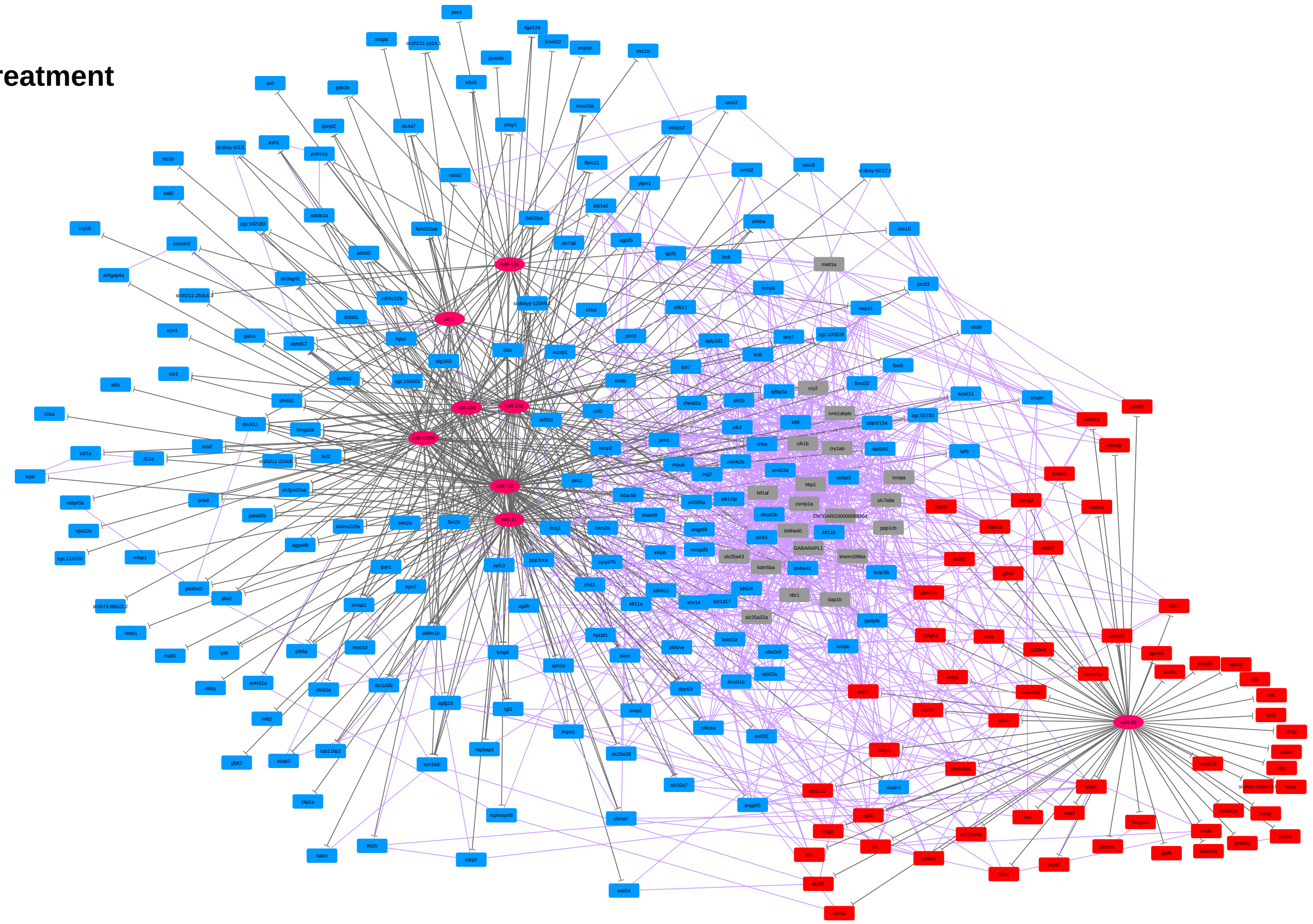

Figure S4. Interaction networks between miRNAs and predicted targets according to AA and IGF1 treatments. The interaction molecular networks show the level of relationship between the differentially expressed genes from the transcriptome possibly targeted by the differentially expressed miRNAs from the microRNAome. miRNAs are represented by pink color, and up- and down-regulated genes are represented respectively by red and blue colors. Gray boxes are genes added by GeneMANIA to improve the association within the network. Gray lines show interaction between miRNAs and genes, and purple lines show interaction between the genes.

IGF1 treatment

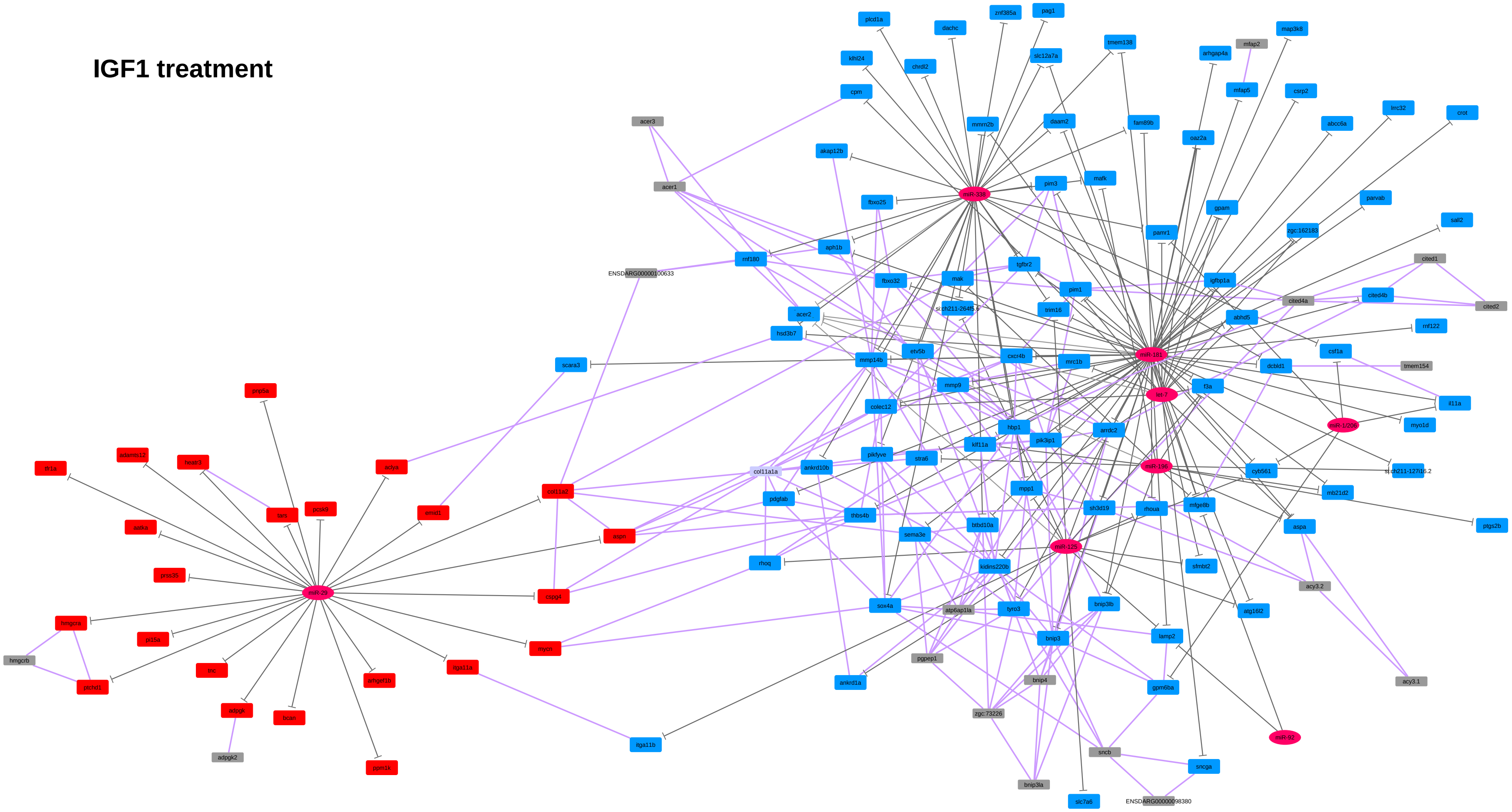

Figure S4. Interaction networks between miRNAs and predicted targets according to AA and IGF1 treatments. The interaction molecular networks show the level of relationship between the differentially expressed genes from the transcriptome possibly targeted by the differentially expressed miRNAs from the microRNAome. miRNAs are represented by pink color, and up- and down-regulated genes are represented respectively by red and blue colors. Gray boxes are genes added by GeneMANIA to improve the association within the network. Gray lines show interaction between miRNAs and genes, and purple lines show interaction between the genes.
