# Supplementary material for: Amino Acids and IGF1 Regulation of Fish Muscle Growth Revealed by Transcriptome and microRNAome Integrative Analyses of Pacu (Piaractus mesopotamicus) Myotubes
Source: Int J Mol Sci. 2022 Jan 21;23(3):1180. doi: 10.3390/ijms23031180 (PMC8835699; doi:10.3390/ijms23031180)
Supplement: Supplementary file 1 [file ijms-23-01180-s001.zip › Figure S5.pdf]

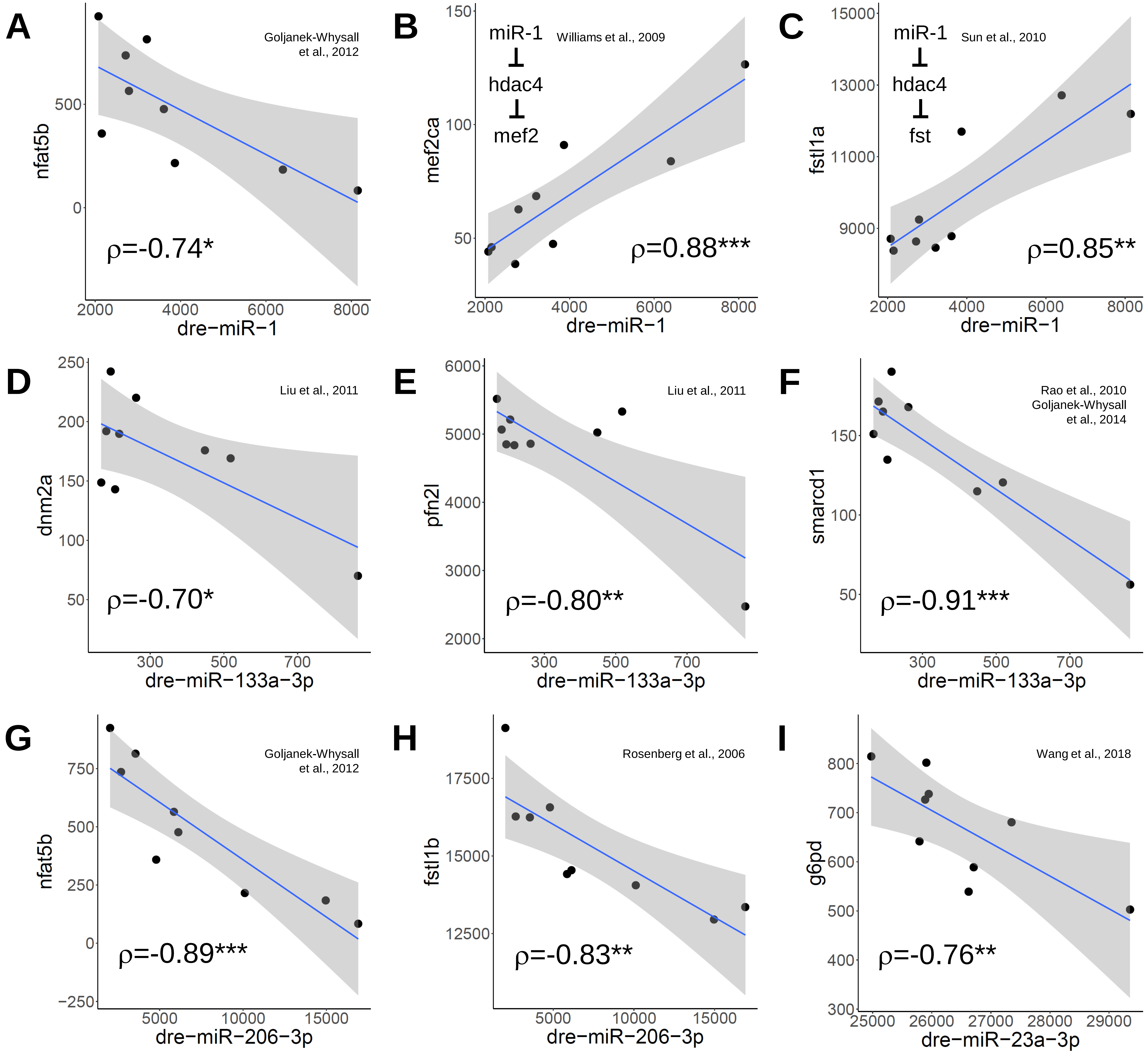

Figure S5. Correlation of validated miRNA-target interaction. Pearson correlation between number of counts for *nfat5b* vs *dre-miR-1* (A), *mef2c* vs *dre-miR-1* (B), *fstl1a* vs *dre-miR-1* (C), *dnm2a* vs *dre-miR-133a-3p* (D), *pfn2l* vs *dre-miR-133a-3p* (E), *smarcd1* vs *dre-miR-133a-3p* (F), *nfat5b* vs *dre-miR-206* (G), *fstl1b* vs *dre-miR-206* (H) and *g6pd* vs *dre-miR-23a* (I). Pearson correlation (ρ) index are indicated in the corners of the plot graphs, and asterisks indicate significant differences between groups: *: P<0.05; **: P<0.01; ***: P<0.001.
