# Supplementary material for: Amino Acids and IGF1 Regulation of Fish Muscle Growth Revealed by Transcriptome and microRNAome Integrative Analyses of Pacu (Piaractus mesopotamicus) Myotubes
Source: Int J Mol Sci. 2022 Jan 21;23(3):1180. doi: 10.3390/ijms23031180 (PMC8835699; doi:10.3390/ijms23031180)
Supplement: Supplementary file 1 [file ijms-23-01180-s001.zip › Figure S6.pdf]

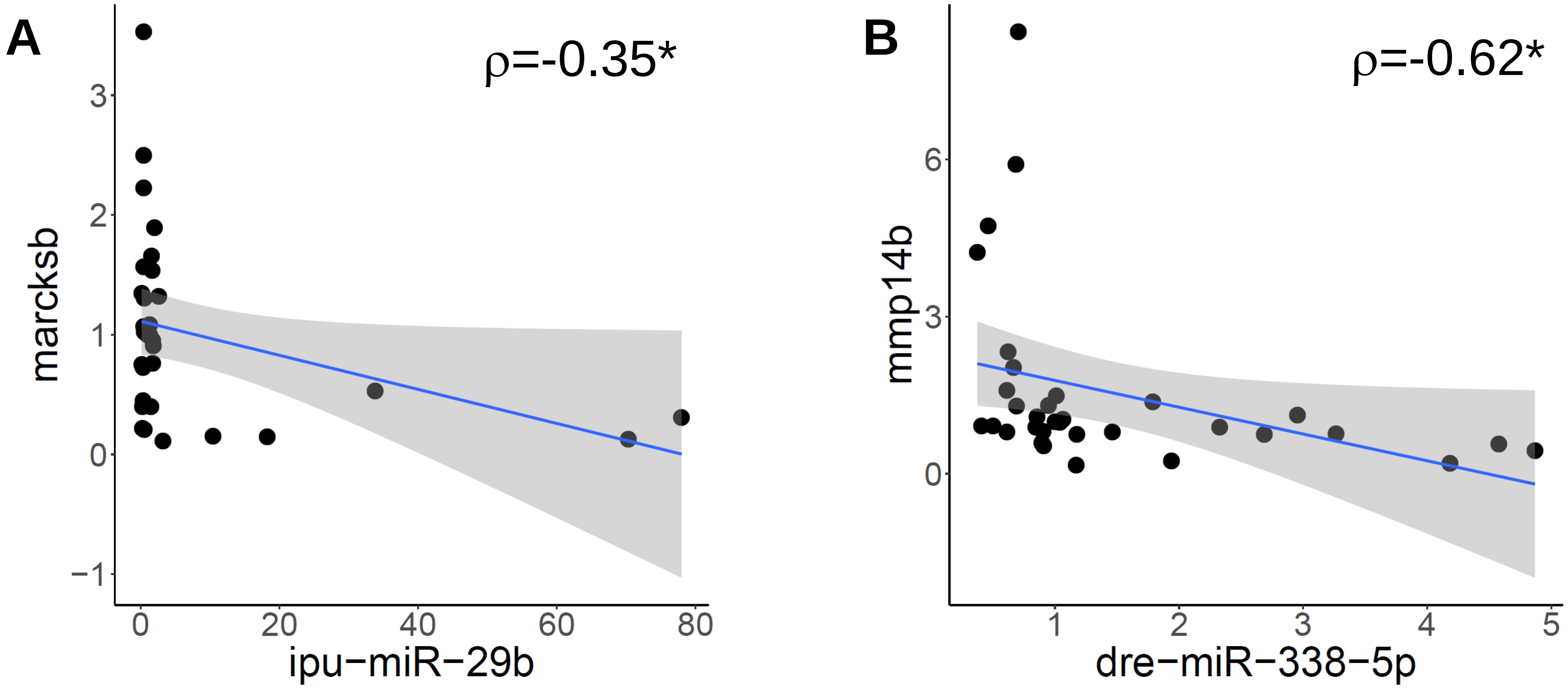

Figure S6. Correlation of *marcksb/miR-29b* and *mmp14b/miR-338-5p* interactions. Pearson correlation between fold-change values for *marcksb* vs *ipu-miR-29b* (A) and *mmp14b* vs *dre-miR-338-5p* (B). Pearson correlation (ρ) index are indicated in the corners of the plot graphs, and asterisks indicate significant differences: *: $P<0.05$.
