# Supplementary material for: Amino Acids and IGF1 Regulation of Fish Muscle Growth Revealed by Transcriptome and microRNAome Integrative Analyses of Pacu (Piaractus mesopotamicus) Myotubes
Source: Int J Mol Sci. 2022 Jan 21;23(3):1180. doi: 10.3390/ijms23031180 (PMC8835699; doi:10.3390/ijms23031180)
Supplement: Supplementary file 1 [file ijms-23-01180-s001.zip › Table S1.pdf]

Table S1. Transcriptome and microRNAome metrics of cultured myotubes from pacu (*Piaractus mesopotamicus*).

Transcriptome Parameters	CTR	AA	IGF1
Reads	70,363,951	69,999,996	76,376,683
Filtered reads	11,555,143	4,342,856	15,004,925
Contigs	176,103		
Annotated contigs	69,932		
Normalized contigs	35,576		
Differentially expressed genes	-----	AA vs CTR<br>1228	IGF1 vs CTR<br>534
Enriched pathways	143		

CTR: Control Group; AA: amino acid Group; IGF1: IGF1 Group.

MicroRNAome Parameters	CTR	AA	IGF1
Reads	40,147,542	40,557,507	44,512,081
Filtered reads	39,319,324	39,554,091	43,712,749
Annotated miRNAs	3,579		
Normalized miRNAs	1,769		
Differentially expressed miRNAs	-----	AA vs CTR<br>11	IGF1 vs CTR<br>8

CTR: Control Group; AA: amino acid Group; IGF1: IGF1 Group.
