# Supplementary material for: Amino Acids and IGF1 Regulation of Fish Muscle Growth Revealed by Transcriptome and microRNAome Integrative Analyses of Pacu (Piaractus mesopotamicus) Myotubes
Source: Int J Mol Sci. 2022 Jan 21;23(3):1180. doi: 10.3390/ijms23031180 (PMC8835699; doi:10.3390/ijms23031180)
Supplement: Supplementary file 1 [file ijms-23-01180-s001.zip › Table S5.pdf]

Table S5. Real time PCR primer sequences for genes and miRNAs. Primers used for qPCR amplification of the *marcksb*, *mycn*, *mmp14b*, *fbxo25*, *tgfbr2*, *ipu-miR-29b* and *dre-miR-338-5p*. Primers were also designed for the reference genes *ppia*, *rpl13* and *U6 snRNA*.

Gene	Primers (5′ to 3′)
marcksb	F: CTCTTTCACTCCCCCAACAA<br>R: GTTGCGGATTGTGTGTTTTG
mycn	F: CTGTTGTGCTGGGACTGAGA<br>R: CAAACTCTTATGGCGGTGGT
mmp14b	F: AAGAACCCTCCCTCACCATC<br>R: CTCCGACTTTGCAGACATCA
tgfbr2	F: AGCTGCTGGGTATCTCTGGA<br>R: TCTATGGCTCTGGTGCTGTG
fbxo25	F: CGTAGGTGAAGTAGCCGCCA<br>R: GGAGAAGTCCAGCCTGTTGA
ppia	F: ATTGTGGTTCGTGAAGTCGC<br>R: CCGCTGGGCAGAGTGATTAT
rpl13	F: ATCAACAGGAAAGTAGCCC<br>R: AGGATGAGTTTGGAGCGGTA
**miRBase ID**	**Mature sequence (5′ to 3′)**
ipu-miR-29b	GCUGAAUUCAGAUGGUGCCAUAGA
dre-miR-338-5p	AACAAUAUCCUGGUGCUGCCUGAGU
U6 snRNA	GTGCTCGCTTCGGCAGCACATATACTAAAATTG<br>GAACGATACAGAGAAGATTAGCATGGCCCCTG<br>CGCAAGGATGACACGCAAATTCGTGAAGCGTT<br>CCATATTTT

ipu, *Ictalurus punctatus*; dre, *Danio rerio*; F, forward; R, reverse.
